# A Single-Step Route to Robust and Fluorine-Free Superhydrophobic Coatings via Aerosol-Assisted Chemical Vapor Deposition

**DOI:** 10.1021/acs.langmuir.3c00554

**Published:** 2023-05-22

**Authors:** Julie Jalila Kalmoni, Frances L. Heale, Christopher S. Blackman, Ivan P. Parkin, Claire J. Carmalt

**Affiliations:** Materials Chemistry Centre, Department of Chemistry, University College London, 20 Gordon Street, London WC1H 0AJ, U.K.

## Abstract

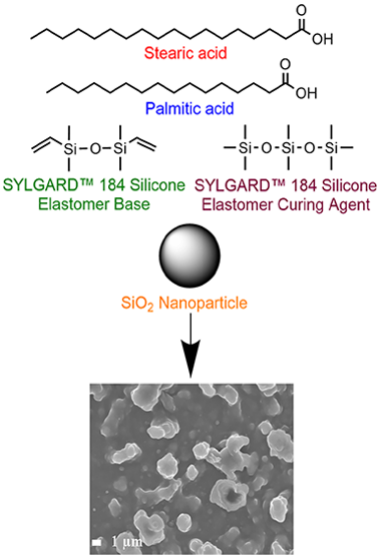

Robust fluorine-free superhydrophobic films were produced from a mixture of two fatty acids (stearic acid and palmitic acid), SiO_2_ nanoparticles, and polydimethylsiloxane. These simple and nontoxic compounds were deposited via aerosol-assisted chemical vapor deposition to provide the rough topography required for superhydrophobicity, formed through island growth of the aggregates. The optimum conditions for well-adhered superhydrophobic films produced films with a highly textured morphology, which possessed a water contact angle of 162 ± 2° and a sliding angle of <5°. Superhydrophobicity was maintained after ultraviolet exposure (14 days at 365 nm), heat treatment (5 h at 300 °C and 5 h at 400 °C), 300 tape peel cycles, and exposure to ethanol and toluene (5 h each).

## Introduction

Superhydrophobicity is a crucial property of materials for many large and small applications in which water adsorption can negatively influence functional performance. Examples include anti-icing coatings on airplanes, tuning the hydrophilicity of materials (e.g., cotton), and creating water resistant fabrics.^1,2^ Superhydrophobicity was first observed in lotus leaves where water droplets roll off of the leaf surface rather than wet it, taking dirt particles with them to improve their functional performance, i.e., light harvesting or leaf health.^3,4^ Superhydrophobicity is induced by the microscopic bumps and air spaces across the leaf, while the waxy cuticle provides the low surface energy required.^5,6^ However, the widespread application of superhydrophobic coatings has been hindered due to their typically poor durability or robustness.^7,8^

The Wenzel and Cassie–Baxter models are the two main models used to describe superhydrophobicity.^9^ Water droplets are more likely to stick to the surface when exhibiting Wenzel behavior as they penetrate protrusions from the surface of the materials. Cassie–Baxter behavior causes the water droplets to roll off or slip as the water droplets interact with the air pockets and peaks of the protrusions.^10,11^ To replicate Cassie–Baxter behavior synthetically, both micro- and nanoscale roughness and a low-surface energy reagent are required.^12^ The latter typically employs the use of toxic fluorinated polymers (due to their high durability and low affinity for water).^13,14^

Nonfluorinated alternatives, such as fatty acid-coated silica or metal nanoparticles, have been used to produce superhydrophobic coatings.^15,16^ The properties of the fatty acids change depending on the length of their hydrophobic carbon chain, as confirmed by Heale et al., who fabricated hydrophobic slurries from innately hydrophilic silica nanoparticles dispersed in solutions of fatty acids.^15,17^ Durable coatings were achieved only in the presence of an adhesive to bind the coating to the substrate. Daneshmand et al. reported stearic acid-coated Al_2_O_3_ nanoparticles dispersed in either ethanol, methanol, or 2-propanol, which were then spray coated onto microscope glass substrates in a two-step process to ultimately produce a superhydrophobic film.^18^ However, the use of a combination of fatty acids to achieve robust superhydrophobic films in a one-step process has not been studied.

The preparation of superhydrophobic films can be categorized into bottom-up approaches, top-down approaches, or a combination of both.^5^ Top-down techniques involve starting with a bulk material, and examples include lithography, templating, and plasma etching.^19−21^ In contrast, bottom-up methods involve using micro- and nanoscale units to build a structure using techniques, such as spin coating, sol–gel, and chemical vapor deposition (CVD).^22−24^

The aim of CVD is to ultimately form a thin solid film on a substrate via reactions in the gas phase to produce different gas-phase precursors.^25,26^ Aerosol-assisted chemical vapor deposition (AACVD) is a type of CVD in which the precursor mixture (dissolved in a solvent) is aerosolised via a nebulizer/ultrasonic humidifier.^24,27^ The carrier gas transports the gaseous precursor to the heated reactor, causing the solvent to evaporate and precursors to react homogeneously or heterogeneously, eventually forming a solid film.^24^

If the precursor mixture is composed of polymers and/or silica nanoparticles, these particles diffuse from the heated carbon block toward the cooler glass top plate (substrate) by Brownian motion, a process described as thermophoresis.^28^ AACVD involves the impaction of particles onto the substrate that are physisorbed, unlike conventional CVD that involves chemisorption.^25,28^ AACVD is easily scalable, producing textured rough surfaces (a requisite for superhydrophobic films), and depends on only the solubility of the precursors (rather than their volatility).^27^

Previous reports on the formation of robust SiO_2_ superhydrophobic thin films via AACVD have mainly involved the use of fluorinated polymers or layer-by-layer depositions to achieve well-adhered films that in some examples are also transparent.^12,29^ Here, durable superhydrophobic thin films were prepared via a single-step AACVD route using SiO_2_ nanoparticles in combination with either nonfluorinated or fluorinated polymers (for comparison). Their durability was achieved by using a combination of stearic acid and palmitic acid, which also engendered a dual-scale roughness, aiding superhydrophobicity. Even though polydimethylsiloxane (PDMS)/SiO_2_ films are superhydrophobic, they are typically non-durable, powdery and can be wiped off the substrate with a tissue.

The water repellency, transparency, adherence, and self-cleaning properties were studied by adjusting the material combinations, loadings, polymer, and deposition temperatures to find the optimum conditions that could allow for the use of nonfluorinated species to create durable superhydrophobic films.

## Experimental Section

Vinyl-terminated PDMS, namely Sylgard-184 Silicone Elastomer Base, and its respective curing agent were purchased from Dow Corning. Aerosil OX50 fumed SiO_2_ nanoparticles (NPs) were purchased from Lawrence Industries. Stearic acid (SA, reagent grade, 95%), palmitic acid (PA, ≥99%), 1*H*,1*H*,2*H*,2*H*-perfluorooctyltriethoxysilane (FAS C_8_, 98%), and ethyl acetate (laboratory grade) were purchased from Sigma-Aldrich. Details of the various combinations used are described in Table 1. N_2_ (99.99%) was supplied by BOC. SiO_2_ barrier-coated float glass was provided by Pilkington NSG and cut into 150 mm × 40 mm × 3 mm pieces for AACVD.

**Table 1:** Summary of the Experimental Conditions Used to Deposit the Superhydrophobic Thin Films via AACVD with a Flow Rate of 1 L/min and the Resulting Water Contact Angles (WCA)

film	FAS C_8_/fatty acid used	mass of FAS C_8_/fatty acid used (g)	temperature of deposition (°C)	total deposition time (min)	WCA (deg)
**PDMS/SiO**_**2**_	–	–	360	40	159 ± 2
**PDMS/SiO**_**2**_**/FAS**	FAS C_8_	0.6	300	90	163 ± 2
**PDMS/SiO**_**2**_**/SA**	SA	0.6	300	90	145 ± 11
**PDMS/SiO**_**2**_**/PA**	PA	0.6	300	90	129 ± 3
**PDMS/SiO**_**2**_**/SA+PA**	SA and PA	0.6	300	90	162 ± 3
**PDMS/SiO**_**2**_**/SA+PA/360**	SA and PA	0.6	360	90	162 ± 2
**PDMS/SiO**_**2**_**/SA+PA/400**	SA and PA	0.6	400	90	129 ± 7
**0.25PDMS/SiO**_**2**_**/0.25(SA+PA)**	SA and PA	0.2	360	40	151 ± 7
**0.5PDMS/SiO**_**2**_**/0.5(SA+PA)**	SA and PA	0.4	360	40	161 ± 2
**0.75PDMS/SiO**_**2**_**/0.75(SA+PA)**	SA and PA	0.6	360	40	162 ± 2
**1.0PDMS/SiO**_**2**_**/1.0(SA+PA)**	SA and PA	0.8	360	40	163 ± 2
**0.5PDMS/SiO**_**2**_**/0.5(SA+PA)/40**^a^	SA and PA	0.4	360	40	161 ± 2
**0.5PDMS/SiO**_**2**_**/0.5FAS/40**	FAS C_8_	0.4	360	40	161 ± 2
**0.5PDMS/SiO**_**2**_**/0.5(SA+PA)/35**	SA and PA	0.4	360	35	161 ± 2
**0.5PDMS/SiO**_**2**_**/0.5(SA+PA)/30**	SA and PA	0.4	360	30	146 ± 14
**0.5PDMS/SiO**_**2**_**/0.5(SA+PA)/25**	SA and PA	0.4	360	25	132 ± 8
**0.5PDMS/SiO**_**2**_**/0.5(SA+PA)/10**	SA and PA	0.4	360	10	112 ± 5

a This film is the same as **0.5PDMS/SiO**_**2**_**/0**.**5(SA+PA)**.

### Synthesis of Superhydrophobic Coatings Using FAS C_8_, Stearic Acid, Palmitic Acid, or a Combination of Both Fatty Acids

Sylgard-184 (0.60 g), its respective curing agent (0.06 g), FAS C_8_ or palmitic acid or stearic acid or a 50:50 mixture of stearic acid and palmitic acid (0.6 g), and ethyl acetate (60 cm^3^) were mixed for 5 min. OX50 SiO_2_ NPs (0.25 g) were then added to the precursor solution and mixed vigorously for an additional 20 min. Subsequently, the film precursor mixture was deposited on barrier-coated float glass in a bottom-down heating configuration where depositions occurred on the glass top plate (substrate). The graphite heating block possessed a Whatmann cartridge heater, which was regulated using a Pt–Rh cartridge heater. This setup was enclosed in a cylindrical quartz tube. Once the desired deposition temperature of 300 °C was reached, a piezoelectric ultrasonic humidifier was used to generate an aerosol that, with the N_2_ carrier gas (1 L min^–1^), traveled through to the heated chamber for 40 min. Then, additional ethyl acetate (30 cm^3^) was added to the precursor mixture and left to deposit for an additional 30 min. Later, more ethyl acetate (20 cm^3^) was added to the precursor mixture but left to deposit for 20 min, with a total deposition time of 1.5 h. At the end of the deposition, the reactor was left to cool under a flow of nitrogen until the temperature was <100 °C. The coated glass top plate (substrate) was then handled in air. The obtained films were denoted as **PDMS/SiO**_**2**_**/FAS**, **PDMS/SiO**_**2**_**/SA**, **PDMS/SiO**_**2**_**/PA**, and **PDMS/SiO**_**2**_**/SA+PA** (where FAS is 1*H*,1*H*,2*H*,2*H*-perfluorooctyltriethoxysilane, SA is stearic acid, and PA is palmitic acid).

### Effect of Deposition Temperature

The procedure described above was repeated using Sylgard-184 (0.60 g), its respective curing agent (0.06 g), a 50:50 mixture of stearic acid and palmitic acid (0.6 g), OX50 SiO_2_ NPs (0.25 g), and ethyl acetate (60 cm^3^) at deposition temperatures of 360 and 400 °C for the film deposition experiments via AACVD. These films were denoted as **PDMS/SiO**_**2**_**/SA+PA/360** and **PDMS/SiO**_**2**_**/SA+PA/400**.

### Effect of Changing the Concentration of All Reagents (except the SiO_2_ NPs)

The concentrations of all of the reagents except the OX50 SiO_2_ NPs were fractionally reduced by 75%, 50%, and 25% for Sylgard-184 (0.30 g), its respective curing agent (0.03 g), and a 50:50 mixture of stearic acid and palmitic acid (0.80 g). The masses and/or volumes of the OX50 SiO_2_ NPs (0.25 g) and ethyl acetate (60 cm^3^) remained unchanged. The deposition time was 40 min with a temperature of 360 °C for all of the films in this study. The films were denoted as **1.0PDMS/SiO**_**2**_**/1**.**0(SA+PA)**, **0.75PDMS/SiO**_**2**_**/0**.**75(SA+PA)**, **0.5PDMS/SiO**_**2**_**/0**.**5(SA+PA)**, and **0.25PDMS/SiO**_**2**_**/0**.**25(SA+PA)**.

### Effect of Deposition Time

The deposition time was varied (40, 35, 30, 25, and 10 min) with the temperature constant at 360 °C using the procedure outlined directly above. The films were denoted as **0.5PDMS/SiO**_**2**_**/0**.**5(SA+PA)/40**, **0.5PDMS/SiO**_**2**_**/0**.**5(SA+PA)/35**, **0.5PDMS/SiO**_**2**_**/0**.**5(SA+PA)/30**, **0.5PDMS/SiO**_**2**_**/0**.**5(SA+PA)/25**, and **0.5PDMS/SiO**_**2**_**/0**.**5(SA+PA)/10**.

### Characterization

The surface morphologies of the films were studied using JEOL JSM-6701F and JEOL JSM-7600F scanning electron microscopes with an accelerating voltage of 5–10 keV. All samples were vacuum sputtered with gold for 10 s prior to imaging to enhance the electrical conductivity of the films. The images were further analyzed using ImageJ version 1.52s. Fourier transform infrared spectroscopy (FT-IR) was performed using a Bruker alpha platinum attenuated total reflection (ATR) instrument, in the range of 400–4000 cm^–1^. Ultraviolet–visible (UV–vis) spectroscopy data were collected using a Shimadzu UV-2700 spectrophotometer in the range of 400–800 nm. X-ray photoelectron spectroscopy data were collected using a Thermo Scientific spectrometer with a monochromated Al K alpha source (8.3381 Å). The peaks were modeled using CasaXPS version 2.3.25, and binding energies were adjusted to adventitious carbon (285.0 eV). The Sq (root-mean-square height) of the films was determined by the Keyence VHX-S750E optical microscope at 1500× magnification, using a Gaussian filter type but no S-filter or L-filter.

A Kruss DSA 25E drop shape analyzer was used to determine the water contact angles (WCAs) of 10 deionized (DI) water droplets of ∼5 μL across the central section of the films and calculated using ADVANCE version 1.14.3. The errors calculated are equivalent to one standard deviation. The sliding angles (SAs) were measured using the tilted drop method with a DI water droplet size of ∼15 μL dispensed close to the middle of the substrate. The stage was already inclined before attempting any measurements. In all instances, the Young–Laplace equation was employed by the software to calculate the size of the angles. The contact angle hysteresis (CAH) was measured by subtracting the advancing contact angle (ACA) from the receding contact angle (RCA) via the sessile drop method.

### Durability

#### Ultraviolet Stability Test

The samples were placed in a sealed box and exposed to ultraviolet (UV) light for 2 weeks with WCAs and SAs measured every 24 h (for the first 4 days and 7 and 14 days thereafter). This was carried out at room temperature with a UV emission wavelength of 365 nm and an intensity of 258 mW/cm^2^ to replicate standard external UV irradiation.

#### Polarity Stability Test

The samples were immersed in two solvents of different polarities, ethanol and toluene, with WCAs measured at 1 h intervals for 5 h. Sliding angles were measured after immersion for 5 h.

#### Heat Stability Test

The samples were placed in a furnace at 300 °C for 5 h and, subsequently, for an additional 5 h at 400 °C. The WCAs were measured after each heat cycle, and SAs were measured at the end of the 10 h exposure.

#### Tape Peel Test

Scotch Magic Tape was stuck to and removed from the film 300 times, with WCAs measured after every 20 cycles and SAs measured after every 100 cycles.

#### Pencil Hardness Test

The Elcometer 501 Pencil Hardness Tester (Elcometer Ltd.) was used to obtain a hardness value based on a standard for ASTM D3363. Pencils with different hardnesses (6H–6B) were inserted into the pencil tester at a 45° angle to the surface and pushed across the film at a constant speed. The softest pencil was used with increasing hardness until a clear line was visible in the coating.

### Self-Cleaning

#### Self-Cleaning Tests

The surfaces of the films were coated with gold glitter, and water droplets were manually dispensed directly onto the surface. Droplets of methylene blue dye were continuously dispensed directly onto the films, at a 20° angle. Photographs before, during, and after the test were taken, and all of the tests were carried out to determine the water repellency and self-cleaning ability of the films.

## Results and Discussion

To investigate the effect of fluorinated versus nonfluorinated reagents for the formation of superhydrophobic coatings, AACVD of an ethyl acetate solution of SiO_2_ NPs, polydimethylsiloxane (PDMS), and a long chain fluoroalkyl or alkyl species, including 1*H*,1*H*,2*H*,2*H*-perfluorooctyltriethoxysilane (FAS C_8_), stearic acid (SA), palmitic acid (PA), or a 50:50 SA/PA mixture, was studied at 300 °C. This combination of reagents enabled the formation of rough surfaces due to the presence of the SiO_2_ NPs, along with hydrophobicity from the PDMS and FAS/SA/PA and durability from the polymer. Altering the long chain fluoroalkyl or alkyl species resulted in a change in hydrophobicity, as described below and given in Table 1. It is interesting to note that using a combination of SA and PA resulted in an increase in water contact angle to 162 ± 3° (compared to 145 ± 11° for SA and 129 ± 3° for PA).

The formation of a superhdrophobic film without using a fluorinated species was achieved via AACVD of PDMS, SiO_2_ NPs, and a 50:50 SA/PA mixture in ethyl acetate at 300 °C (**PDMS/SiO**_**2**_**/SA+PA** film). This combination of reagents was therefore chosen to investigate the effect of changing the deposition temperature on the resulting film. These studies involved investigating the deposition of the same reagent mix at 360 and 400 °C, affording **PDMS/SiO**_**2**_**/SA+PA/360** and **PDMS/SiO**_**2**_**/SA+PA/400** films, respectively. The film deposited at 360 °C produced the most well-adhered superhydrophobic film, and hence, a temperature of 360 °C was used in all subsequent depositions. First, this involved studying the effect of the loading of PDMS and fatty acids in the precursor mixture. The concentrations of all of the reagents except the SiO_2_ NPs were fractionally reduced by 25%, 50%, and 75%, relative to the unchanged film, producing ***x*PDMS/SiO**_**2**_**/*x*(SA+PA)** films (*x* = 0.25, 0.5, 0.75, and 1.0), as shown in Table 1. The final study involved varying the deposition time from the original 40 min to 35, 30, 25, and 10 min with the temperature kept constant at 360 °C and the same reagents (PDMS, a 50:50 SA/PA mixture, and SiO_2_ NPs in ethyl acetate), as shown in Table 1. The variation of the deposition time was investigated at shorter deposition times rather than between 40 and 90 min because the films deposited at 40 and 90 min were very similar with similar WCAs. All resulting coatings were characterized using a range of techniques, including X-ray photoelectron spectroscopy (XPS), scanning electron microscopy (SEM), and FT-IR, and their functional properties were tested.

XPS was used to understand the surface chemistry of the resulting films, and the adventitious carbon peak at 285.0 eV was used as a charge reference. The XPS survey spectra of all films were consistent, indicating the presence of only C, O, and Si (except for the **PDMS/SiO**_**2**_**/FAS** film that also contains fluorine as expected). The XPS survey spectrum of a representative film, namely **PDMS/SiO**_**2**_**/SA+PA**, is shown in Figure S1. The XPS spectra for a **PDMS/SiO**_**2**_**/FAS** film are also given in Figure S2.

**Figure 1:**
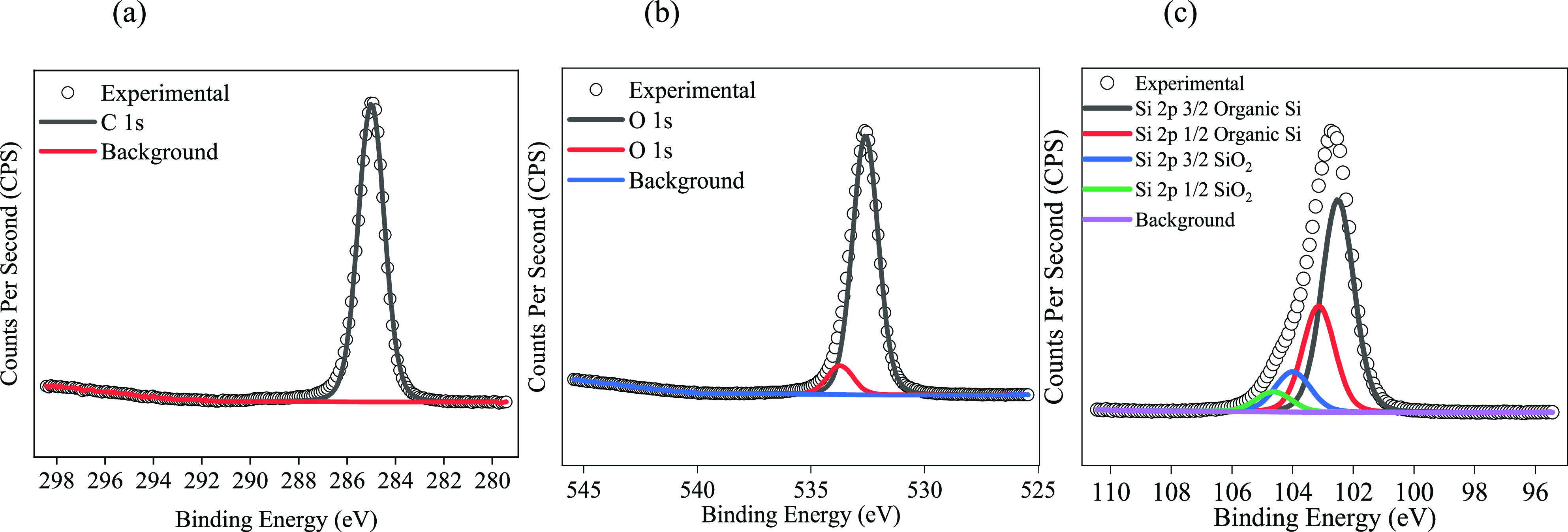
X-ray photoelectron data for the **PDMS/SiO**_**2**_**/SA+PA** film showing the (a) C 1s, (b) O 1s, and (c) Si 2p spectra.

The deconvoluted C, O, and Si XPS spectra for all of the films were consistent, and a representative example is shown in Figure 1 for a **PDMS/SiO**_**2**_**/SA+PA** film. The C 1s spectra (Figure 1a) contain a peak at 285.0 eV, indicative of a C–O bridge between the Si–O bond of the NP and the carbon chain of the fatty acid, leading to the conclusion that the fatty acid attaches to the SiO_2_ NP via the carboxyl group.^30^Figure 1b (O 1s) confirms the presence of SiO_2_ (oxygen bound to Si) and organic C–O due to the peaks at 532.6 and 533.6 eV, respectively.^17,31^Figure 1c (Si 2p) proves the presence of organic silicon (102.5 eV) and SiO_2_ (Si bound to O) at 104.0 eV, which is verified by the fact that O is bound to Si as in the O 1s spectrum, represented by the peak at 532.6 eV.^32^ All peaks are in line with the literature.

**Figure 2:**
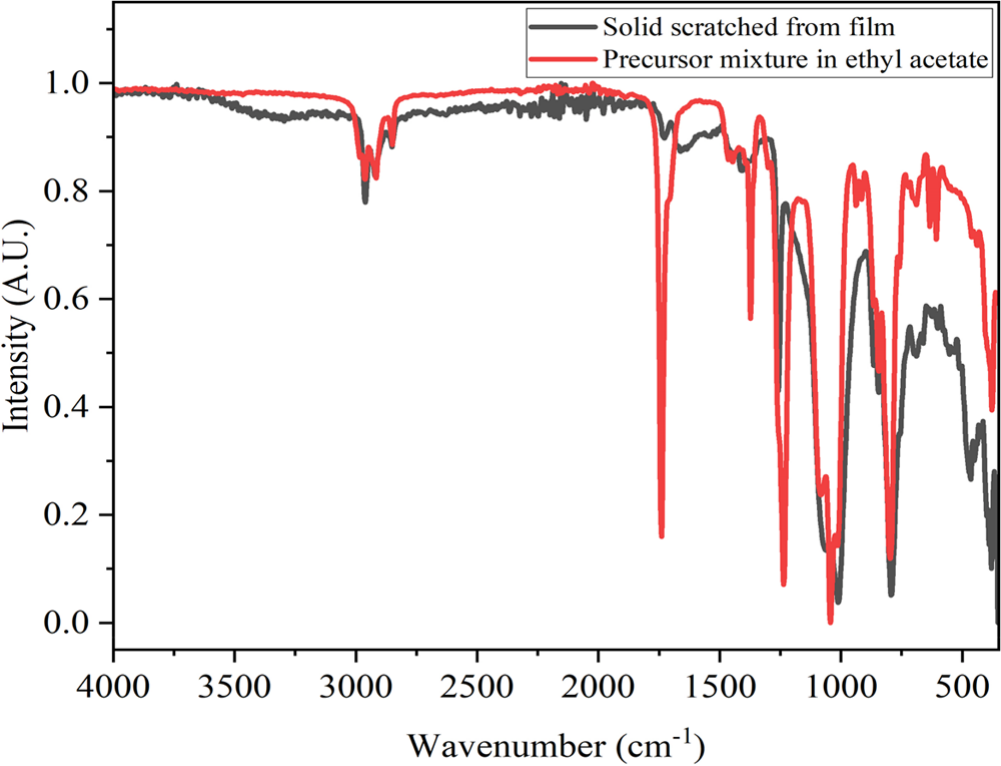
Fourier transform infrared (FT-IR) spectrum of the precursor mixture used to deposit the **PDMS/SiO**_**2**_**/SA+PA** film and the film itself (as a solid).

The FT-IR spectra of all films incorporating fatty acids (SA, PA, or SA and PA) were similar regardless of the deposition conditions used, and a representative spectrum is shown in Figure 2. All films consisted of similar precursor mixtures (except when using FAS), with slight changes to the concentration of reagents or deposition conditions. Therefore, the individual peaks may pertain to more than one reagent due to the films having the same elements, C, H, O, and Si (except **PDMS/SiO**_**2**_**/FAS**), and no contaminants, as confirmed by XPS. The FT-IR spectra of the starting materials (OX50 SiO_2_ NPs, PDMS, and its respective curing agent and the precursor mixture in ethyl acetate used to deposit the film) are presented in Figure S3. The FT-IR spectrum of the **PDMS/SiO**_**2**_**/FAS** film is shown in Figure S4.

All films displayed a strong stretch at ∼1010 cm^–1^ that could be assigned to the Si–O–Si asymmetric stretching vibrations. The sharp stretch at ∼2960 cm^–1^ was due to the Si-CH_3_ group, specifically the sp^3^ C–H asymmetric stretch, indicative of the terminal -CH_3_ groups of the PDMS. Similarly, all films contained at least one medium-intensity peak at 2910 or 2845 cm^–1^, indicative of the CH_2_ asymmetric or symmetric bonds, respectively. This could be of the PDMS or CH_2_ of the fatty acids.^33^ The strong peak at ∼1260 cm^–1^ corresponded to the sp^3^ C–H deformation.^34^ The medium and strong peaks at 870 and 790 cm^–1^, respectively, were the stretching vibrations of CH_3_. The strong/broad peaks at ∼1040 and ∼790 cm^–1^ are indicative of the Si–O–Si asymmetric and symmetrical stretches, respectively, of PDMS and fumed SiO_2_ NPs.^35,36^

A small but sharp carboxylic acid C=O peak at approximately 1727 cm^–1^ and the presence of the -OH stretch of the carboxyl group indicates the presence of the carboxyl groups of stearic acid and palmitic acid. Similarly, the positioning of this -OH group suggests that the fatty acids do not dimerize but remain as aliphatic chains.^37^ Nevertheless, all peaks present were in close agreement with the literature.

**Figure 3:**
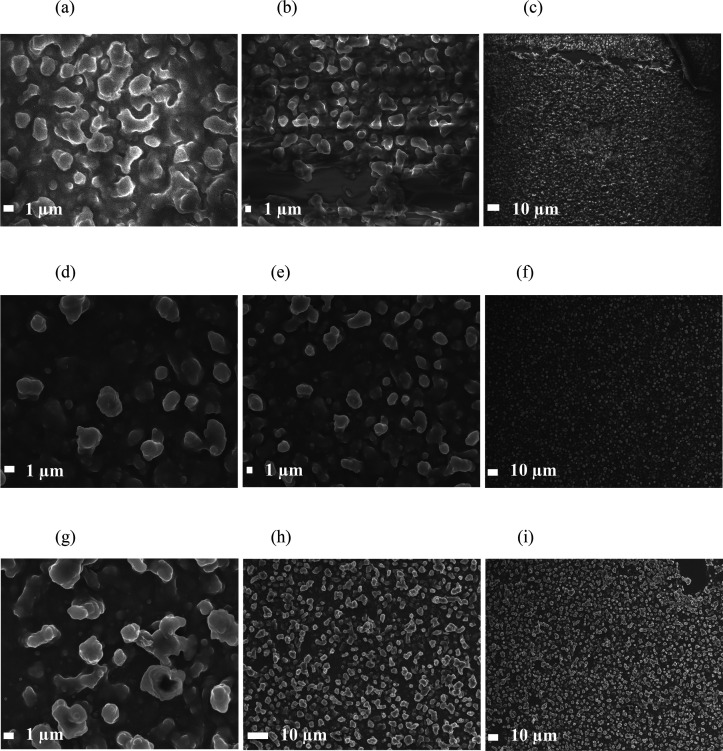
SEM images of films. Images a–c depict the morphology of **PDMS/SiO**_**2**_**/SA** (SiO_2_ NPs coated with stearic acid). Images d–f represent **PDMS/SiO**_**2**_**/PA** (SiO_2_ NPs coated with palmitic acid). Images g–i depict **PDMS/SiO**_**2**_**/SA+PA** (SiO_2_ NPs coated with a 50:50 stearic acid/palmitic acid mixture).

High-magnification SEM images (Figure 3) indicated the presence of an interconnected network of particles that were nonspherical and nonuniform, which was greater when using a combination of fatty acids (**PDMS/SiO**_**2**_**/SA+PA** film). For example, SEM of the film using only palmitic acid (**PDMS/SiO**_**2**_**/PA** film) showed lower variability in the distance between the ends of the interconnected networks that ranged from 1.3 to 6.2 μm. In contrast, the size range of the particles for the **PDMS/SiO**_**2**_**/SA+PA** film varied from 1.2 to 9.1 μm with the lower limit relating to individual particles and the larger limit being the distance between the ends of the interconnected network.

Upon investigation of the WCA, it was found that coating the SiO_2_ NPs with a single fatty acid (**PDMS/SiO**_**2**_**/SA** and **PDMS/SiO**_**2**_**/PA** films) resulted in variable hydrophobicity (WCAs of 145 ± 11° and 129 ± 3°) and not superhydrophobicity. However, using a combination of two fatty acids (**PDMS/SiO**_**2**_**/SA+PA** film) led to superhydrophobicity across the entire film (WCA of 165.3 ± 1.6°), which was comparable to the WCAs from films deposited using the fluorinated polymer (163 ± 2°). The differences in WCA are consistent with the differences in the observed morphologies of the films. The SEM images (Figure 3) confirmed that the superhydrophobic films consisted of nano- and microscale particles of a range of sizes to create a textured morphology. An increase in the number of particles of different sizes led to greater roughness and hence increased the superhydrophobicity of the film confirmed by the Sq values of **PDMS/SiO**_**2**_**/SA**, **PDMS/SiO**_**2**_**/PA**, **PDMS/SiO**_**2**_**/SA+PA**, and **PDMS/SiO**_**2**_**/FAS** that are 0.69, 0.27, 1.32, and 1.35 μm, respectively. This was because air spaces could penetrate underneath the water droplet and hence increase the WCA. Some of the particles agglomerated to form microparticles or larger interconnected networks.

**Figure 4:**
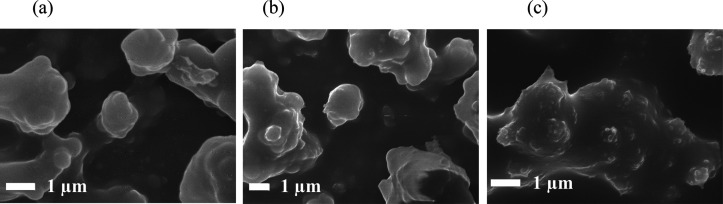
SEM images of the morphology of all films produced from PDMS and SiO_2_ NPs coated with a 50:50 stearic acid/palmitic acid mixture but at (a) 300, (b) 360, and (c) 400 °C.

**Figure 5:**
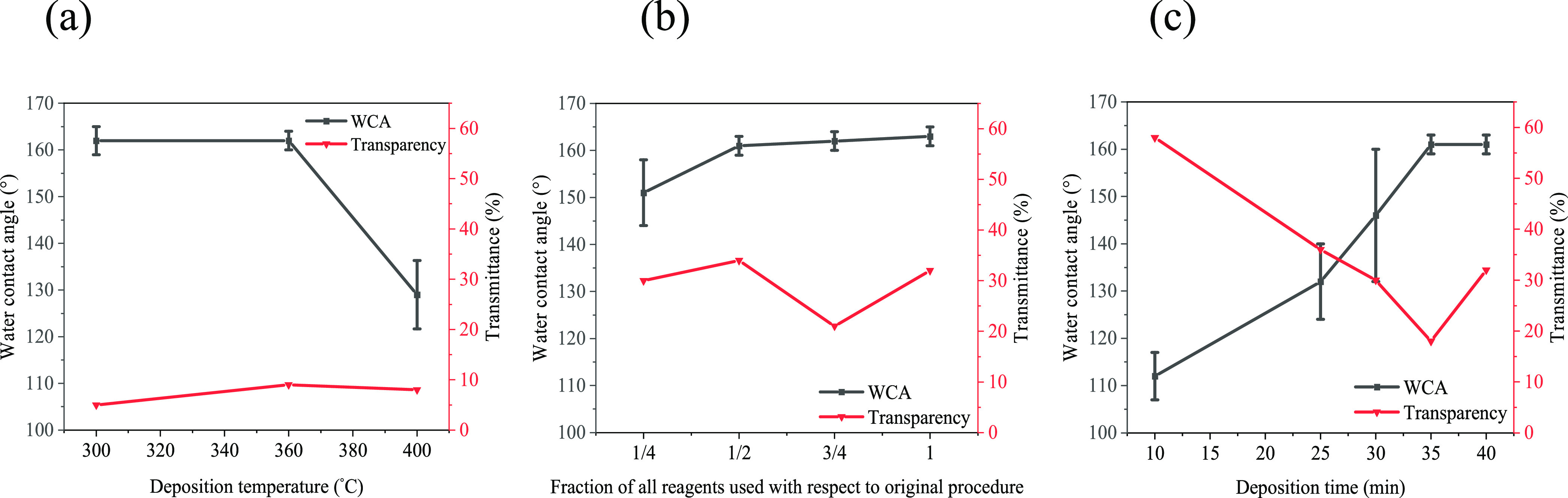
Water contact angles for films deposited from PDMS, SiO_2_ NPs, and a 50:50 SA/PA mixture (a) for the temperature study at 300, 360, and 400 °C, (b) by varying the concentration of ***x*PDMS/SiO**_**2**_**/*x*(SA+PA)** reagents (*x* = 0.25, 0.5, 0.75, and 1), and (c) by varying the deposition time (10, 25, 30, 35, and 40 min).

As AACVD of PDMS, SiO_2_ NPs, and a 50:50 SA/PA mixture in ethyl acetate at 300 °C resulted in the formation of superhydrophobic films, this combination of reagents was used to investigate the effect of increasing the deposition temperature on the microstructure of the resulting film. The high-magnification SEM images for the films deposited at 300, 360, and 400 °C (Figure 4) show that the films formed via island growth and the presence of spherical particles on the large, interconnected network of SiO_2_ particles. In addition to the microparticles seen in all films, films grown at 360 and 400 °C have smaller particles on the elongated agglomerated structures, which were present in larger quantities in the latter. The small particles were more significant in the film deposited at 400 °C, which may have resulted due to fast evaporation of the precursor solvent (ethyl acetate), or the higher temperature could have led to faster curing of the Sylgard-184, leading to the formation of smaller microparticles.^12^ Increasing the deposition temperature from 300 to 360 °C resulted in similar Sq values (1.32 and 1.24 μm) and no change in the WCA (Figure 5). However, increasing the temperature to 400 °C led to hydrophobicity, with a WCA of 129 ± 7°, which may be due to a decrease in surface roughness (Sq = 0.34 μm).

The effect of changing the concentration of the reagents in the precursor mixture was investigated. The concentrations of all of the reagents except the SiO_2_ NPs were fractionally reduced by 25%, 50%, and 75% relative to the unchanged film, producing ***x*PDMS/SiO**_**2**_**/*x*(SA+PA)** films (*x* = 0.25, 0.5, 0.75, and 1.0), as shown in Table 1. It was hypothesized that decreasing the concentration but keeping the deposition time the same may help in terms of decreasing the variability of the resulting coatings. The films deposited with different concentrations of reagents all had XPS, FT-IR, and SEM images similar to those of the films described above, as expected. A comparison of the WCAs for these films (Figure 5c and Table 1) shows little change in the WCA upon alteration of the concentration of the precursor mixture until the lowest concentration (25% of the original amount), which was ∼12° lower. This illustrates how the presence of the PDMS can enhance the superhydrophobicity. There was little change in transparency among these films; however, the lowest transmittance was observed for the **0.75PDMS/SiO**_**2**_**/0.75(SA+PA)** film. This could be because film thickness can be difficult to control via AACVD, and hence, different areas of the resulting film may have different thicknesses.

The deposition time was varied (40, 35, 30, 25, and 10 min) to explore its effect on the film’s hydrophobicity, thickness, and transparency. The temperature was kept constant at 360 °C, and PDMS, a 50:50 SA/PA mixture, OX50 SiO_2_ NPs, and ethyl acetate were used in each experiment. Analysis of the resulting films (XPS and FT-IR) shows that they were similar to the films described above. However, the SEM images indicate that as the deposition time was reduced, this led to less coverage and more porosity, which can be used to explain changes in hydrophobicity.

As shown in Figure 5c, a link between reducing the deposition time and the level of hydrophobicity was observed. The longer the deposition time, the more hydrophobic the film, with water contact angles ranging from hydrophobic (WCA of 112°) at the shortest deposition time (10 min) to superhydrophobic (WCA of 161°) at 35 min.

Superhydrophobicity and transparency are competing variables. As the WCA increases through a rougher and more complex morphology, the transparency decreases, which is evident in all of the films. A lower transmittance was observed for the **PDMS/SiO**_**2**_**/SA** (5%) and **PDMS/SiO**_**2**_**/SA+PA** (5%) films, relative to the **PDMS/SiO**_**2**_**/PA** (12%) film, and this is supported by the SEM images (Figure 3), which shows denser particles making the penetration of visible light difficult. The slight variations in transmittance of the films across the studies could be due to differences in the size of the particles, hence, their scattering with visible light, density of particles per unit area, and film thickness. The transmittance varied with deposition time due to the thickness of the film. Reducing the deposition time to 10 min created a thin film with a %*T* of ∼60, although the resulting film was hydrophobic and not superhydrophobic.

### Functional Testing

The optimum film of all of the different studies was **0**.**5PDMS/SiO**_**2**_**/0**.**5(SA+PA)/40** deposited at 360 °C because it was a well-adhered film with a high WCA. Therefore, this film was chosen to investigate the functional properties. For the purpose of comparison, the **0**.**5PDMS/SiO**_**2**_**/0**.**5FAS/40** film (a fluoroalkylsilane equivalent) was also studied. Images of the microstructure of the **0**.**5PDMS/SiO**_**2**_**/0**.**5FAS/40** film are shown in Figure S5. A comparison of the WCAs, CAH, and SAs shows that films deposited with SA and PA were superhydrophobic (Tables 1 and 2). The **0**.**5PDMS/SiO**_**2**_**/0**.**5(SA+PA)/40** film had a WCA of 163 ± 1°, which was similar to that of **0**.**5PDMS/SiO**_**2**_**/0**.**5FAS/40** (161 ± 2°); however, robustness tests proved that combining fatty acids contributed to the overall durability of the films.

**Table 2:** Summary of Water Contact Angles, Sliding Angles, Contact Angle Hysteresis, and Root-Mean-Square Heights for the **0**.**5PDMS/SiO**_**2**_**/0**.**5(SA+PA)/40** and **0**.**5PDMS/SiO**_**2**_**/0**.**5FAS/40** Films (with and without FAS C_8_, respectively)

film	FAS C_8_/fatty acid used	deposition time (min)	water contact angle (deg)	sliding angle (deg)	contact angle hysteresis (deg)	Sq (μm)
**0.5PDMS/SiO_2_/0.5(SA+PA)/40**	50:50 stearic acid/palmitic acid mixture	40	163 ± 1	4 ± 1	14 ± 4	0.60
**0.5PDMS/SiO_2_/0.5FAS/40**	FAS C_8_	40	161 ± 2	4 ± 1	20 ± 9	0.66

During the sliding angle tests, the water droplet (∼15 uL) rolled off at 4° indicating water repellency. The high-contact angle hysteresis (CAH) for both films (>10°) indicates Wenzel type behavior (a homogeneous regime) as the water droplet sticks to the surface and penetrates the protrusions, limiting the ability of the water droplet to move across a horizontal surface.^10,11,38^

**Figure 6:**
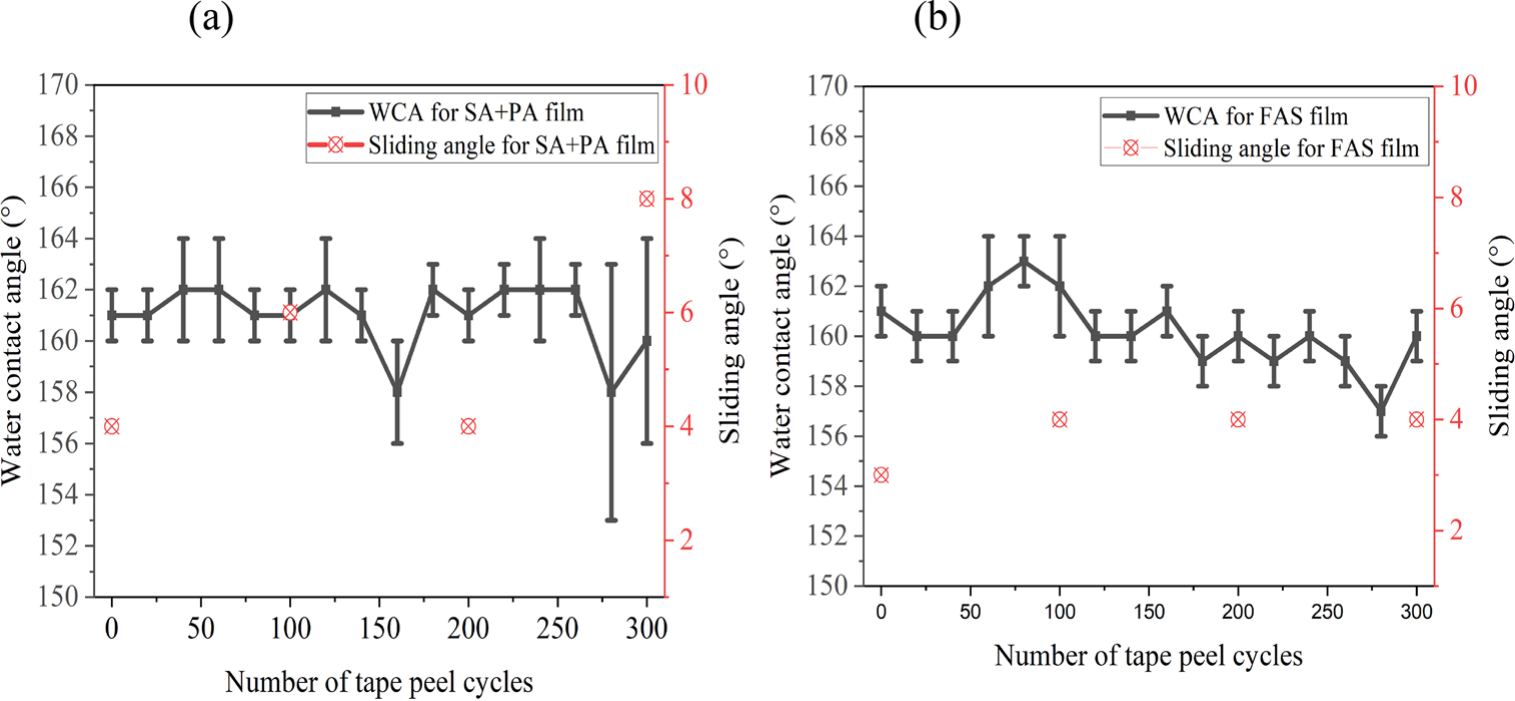
Water contact angles and sliding angles during 300 tape peel cycles for (a) **0**.**5PDMS/SiO**_**2**_**/0**.**5(SA+PA)/40** and (b) **0**.**5PDMS/SiO**_**2**_**/0**.**5FAS/40**.

Several methods were pursued to determine the robustness of films such as UV irradiation, exposure to solvents of different polarities, and adhesive tape peel cycles. The adhesion of coatings to the glass substrates was evaluated via the tape peel test. Even after 300 cycles of the tape peel test, the WCAs of both films [with and without the fluoroalkylsilane (Figure 6)] were >150°, and sliding angles were <10°, indicating strong adhesion of the film to the substrate. For comparison, a **PDMS/SiO**_**2**_ film underwent 20 cycles of the tape peel test. Before the test, it had a water contact angle of 159 ± 2°, but after 20 cycles, this became 147 ± 2°, indicating hydrophobicity and poor durability.

**Figure 7:**
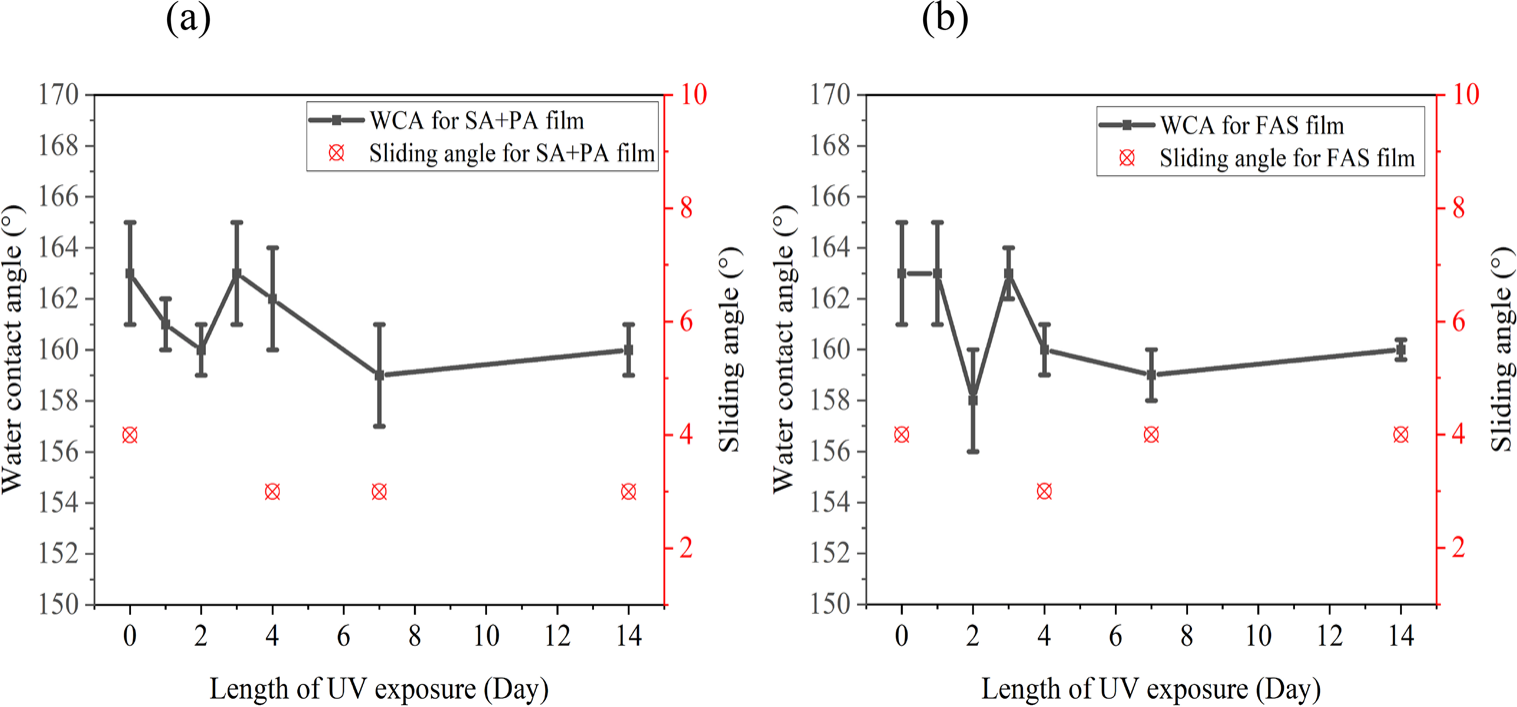
Water contact angles and sliding angles during UV irradiation for 14 days of (a) **0.5PDMS/SiO**_**2**_**/0.5(SA+PA)/40** with a 50:50 SA/PA mixture and (b) **0.5PDMS/SiO**_**2**_**/0.5FAS/40** with FAS C_8_.

Figure 7 highlights the stability of **0**.**5PDMS/SiO**_**2**_**/0**.**5(SA+PA)/40** and **0**.**5PDMS/SiO**_**2**_**/0**.**5FAS/40** films upon exposure to UV irradiation, which is known to oxidize the organic components, creating hydrophilic groups.^39^ Nevertheless, even after UV exposure (λ = 365 nm), both films retained their superhydrophobicity throughout the 14-day cycle with WCAs of >155° and some increase in sliding angle (although it is still <10°). Minimal changes are due to the chemical composition of the films; SiO_2_ NPs and fatty acids are not photoactive and hence resistant to UV radiation.

Table 3 gives the results of heat stability tests that showed both films maintained their superhydrophobicity following exposure at 300 °C for 5 h. After exposure at 400 °C for 5 h, the WCA for **0.5PDMS/SiO**_**2**_**/0.5FAS/40** decreased to 159°. The opposite was observed for **0.5PDMS/SiO**_**2**_**/0.5(SA+PA)/40**, with WCAs reaching 170°, potentially due to the evaporation of excess ethyl acetate, chain diffusion of the hydrophobic carbon chain to the film’s surface, or formation of organic compounds on the surface.^40^

**Table 3:**
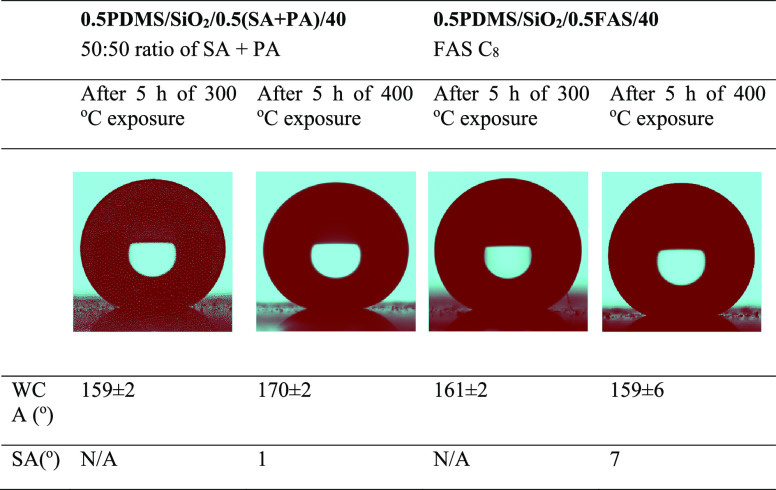
Images, Water Contact Angle Measurements, and Sliding Angle Measurements for the **0.5PDMS/SiO**_**2**_**/0.5(SA+PA)/40** and **0.5PDMS/SiO**_**2**_**/0.5FAS/40** Films after Heating at 300 °C for 5 h and 400 °C for an Additional 5 h

**Figure 8:**
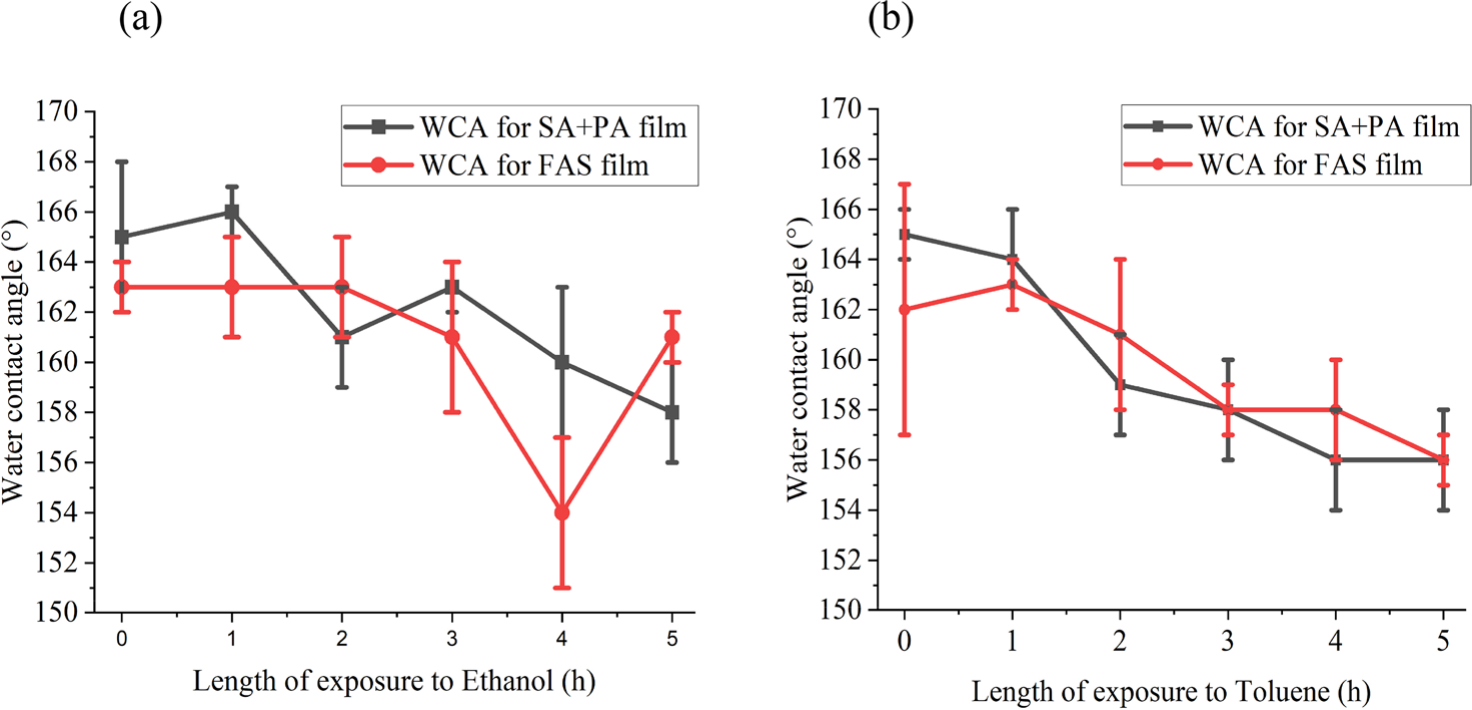
Water contact angles for **0.5PDMS/SiO**_**2**_**/0.5(SA+PA)/40** and **0.5PDMS/SiO**_**2**_**/0.5FAS/40** films during exposure for 5 h to (a) ethanol and (b) toluene.

Upon exposure to solvents of contrasting polarity (ethanol and toluene) for 5 h, the measured sliding angles were largely unchanged for both **0.5PDMS/SiO**_**2**_**/0.5(SA+PA)/40** and **0.5PDMS/SiO**_**2**_**/0.5FAS/40** with the maximum change being a 1° reduction in sliding angle for **0.5PDMS/SiO**_**2**_**/0.5FAS/40** after exposure to toluene for 5 h (Figure 8). There was a <10° reduction in WCA in both films, which is likely due to strong physical attractions between the fatty acids coating the NPs and glass substrate in addition to the low surface energy of the PDMS and fatty acids.

**Figure 9:**
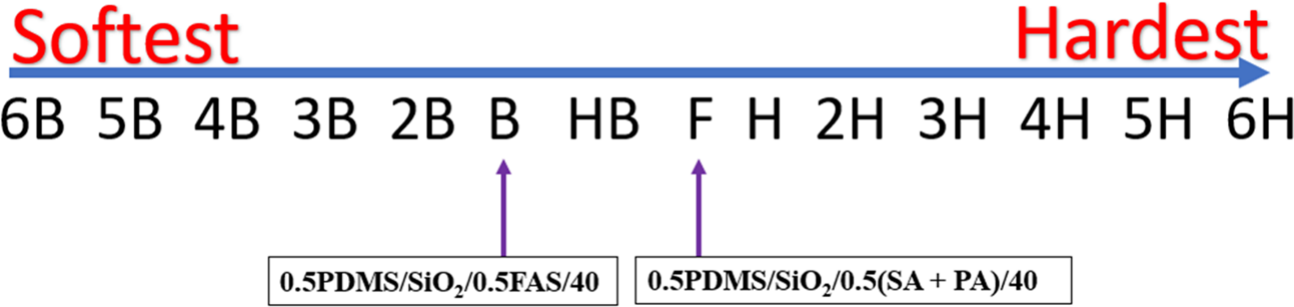
Measures of the hardness for **0.5PDMS/SiO**_**2**_**/0.5(SA+PA)/40** and **0.5PDMS/SiO**_**2**_**/0.5FAS/40** films.

The ability of the films to tolerate hardness was measured using an elcometer (Figure 9) with the robustness of the **0.5PDMS/SiO**_**2**_**/0.5FAS/40** film being less than that of the **0.5PDMS/SiO**_**2**_**/0.5(SA+PA)/40** film due to the film’s microstructure. Nevertheless, both films displayed reduced mechanical resistance relative to films with similar compositions deposited via other methods as AACVD involves the physisorption of the films onto the glass top plate (substrate) rather than chemisorption.^25,28^

**Figure 10:**
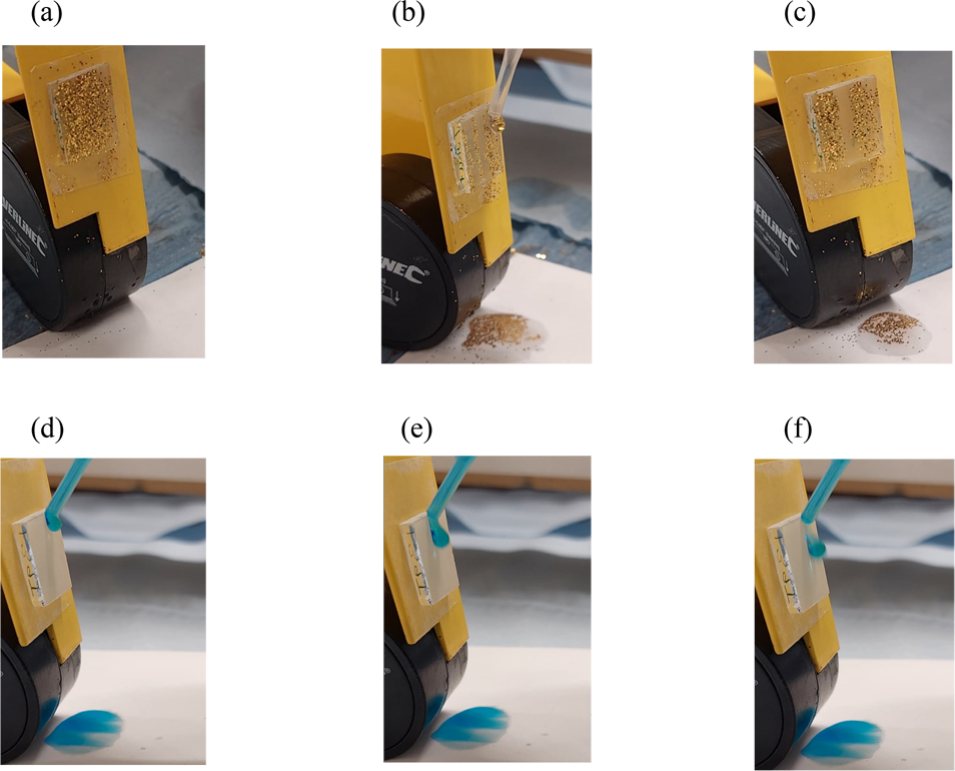
Self-cleaning ability of the **0.5PDMS/SiO**_**2**_**/0.5(SA+PA)/40** film using gold glitter: (a–c) methylene blue dye and (d–f) both at a tilted angle of 20°.

Self-cleaning tests with **0.5PDMS/SiO**_**2**_**/0.5(SA+PA)/40** were performed at a tilt angle of 20°. Its self-cleaning ability was visualized by coating the surface with gold glitter and pipetting water droplets directly onto the film (Figure 10). As shown in Figure 10a, the water droplets slid off, readily clearing the glitter in its path, demonstrating its self-cleaning ability. In a stain test, multiple water droplets of methylene blue dye were pipetted on the surface to determine if it stained the films. The resultant films were dry and unstained due to the water’s high surface tension and the coating’s low surface energy.

## Conclusion

In this study, robust fluorine-free superhydrophobic films of PDMS, its respective curing agent, SiO_2_ NPs, and a 50:50 SA/PA mixture were deposited via AACVD using a one-pot precursor mixture. All reagents were nontoxic, successfully incorporated into the films, and had water contact angles >150° regardless of the deposition conditions. The best film, deposited at 360 °C for 40 min, displayed excellent mechanical durability after 300 tape peel cycles and retained superhydrophobicity after exposure for 5 h to solvents of contrasting polarities, heat exposure at 400 °C, and UV irradiation for 14 days. The film also demonstrated self-cleaning abilities. Previous research has demonstrated that when using AACVD, a fluoroalkylsilane or multiple depositions are required to produce superhydrophobic coatings.^13,29,41^ This study has shown that a combination of two fatty acids can contribute to superhydrophobicity and increased robustness, hence providing a facile new route for producing nontoxic superhydrophobic coatings. Further work could involve improving the transparency of the films.

## Supporting Information Available

X-ray photoelectron survey spectra and FT-IR spectra of films with SA/PA mixtures and FAS and SEM images of **0.5PDMS/SiO_2_/0.5FAS/40** (PDF)
