# Supplementary material for: A Single-Step Route to Robust and Fluorine-Free Superhydrophobic Coatings via Aerosol-Assisted Chemical Vapor Deposition
Source: Langmuir. 2023 May 22;39(22):7731–40. doi: 10.1021/acs.langmuir.3c00554 (PMC10249402; doi:10.1021/acs.langmuir.3c00554)
Supplement: Supplementary file 1 — la3c00554_si_001.pdf [file la3c00554_si_001.pdf]

Supporting Information

A Single Step Route to Robust and Fluorine-free Superhydrophobic Coatings via Aerosol Assisted Chemical Vapour Deposition

Julie Jalila Kalmoni, Frances L. Heale, Christopher S. Blackman, Ivan P. Parkin and Claire J. Carmalt*

*Corresponding author

Materials Chemistry Centre, Department of Chemistry, University College London, 20 Gordon Street, London WC1H 0AJ, UK; c.j.carmalt@ucl.ac.uk

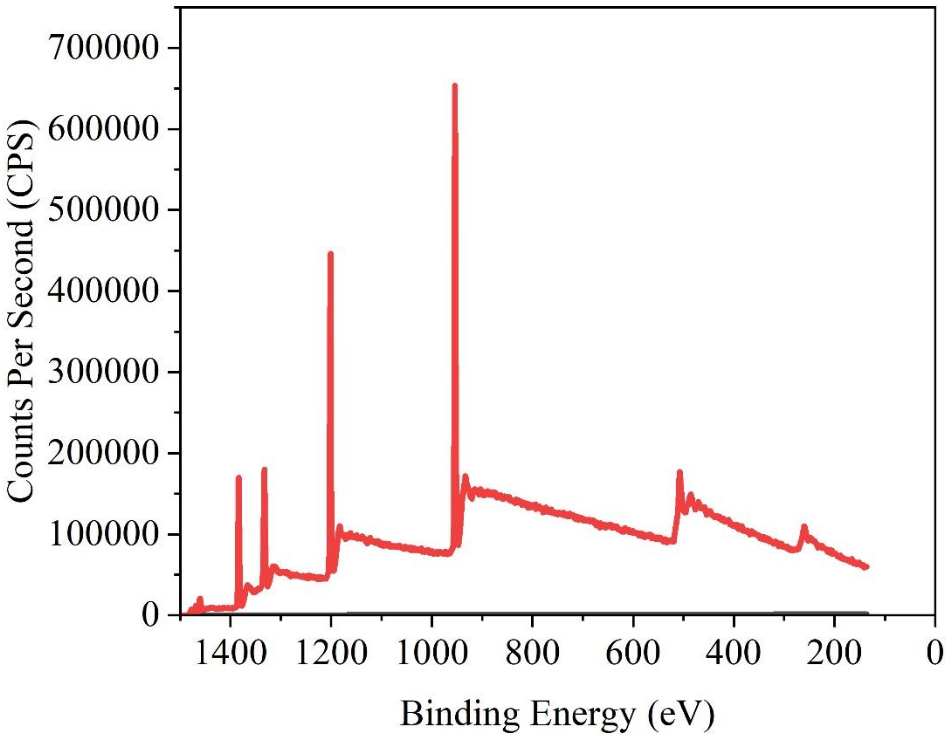

Figure S1. X-ray photoelectron survey spectrum for the film **PDMS/SiO_2/SA+PA.**

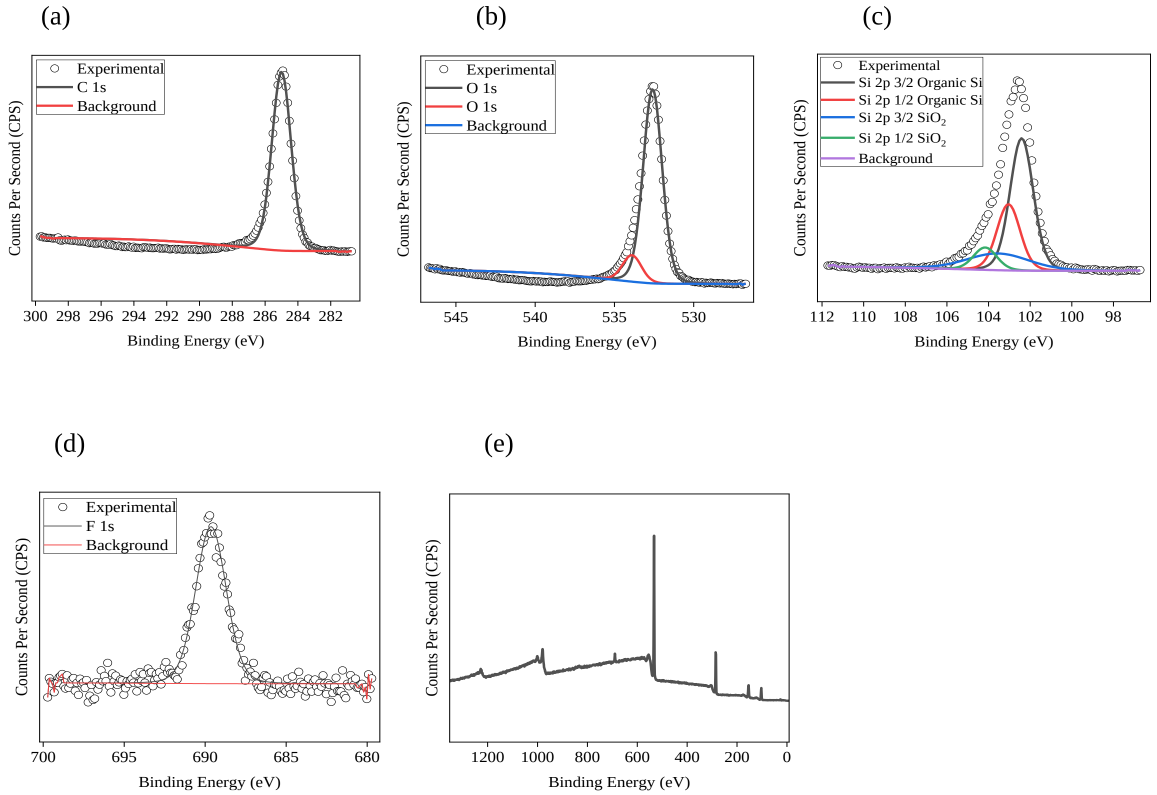

Figure S2. X-ray photoelectron data for the film **PDMS/SiO_2/FAS** showing the (a) C 1s, (b) O 1s, (c) Si 2p, (d) F 1s spectra and (e) survey spectrum.

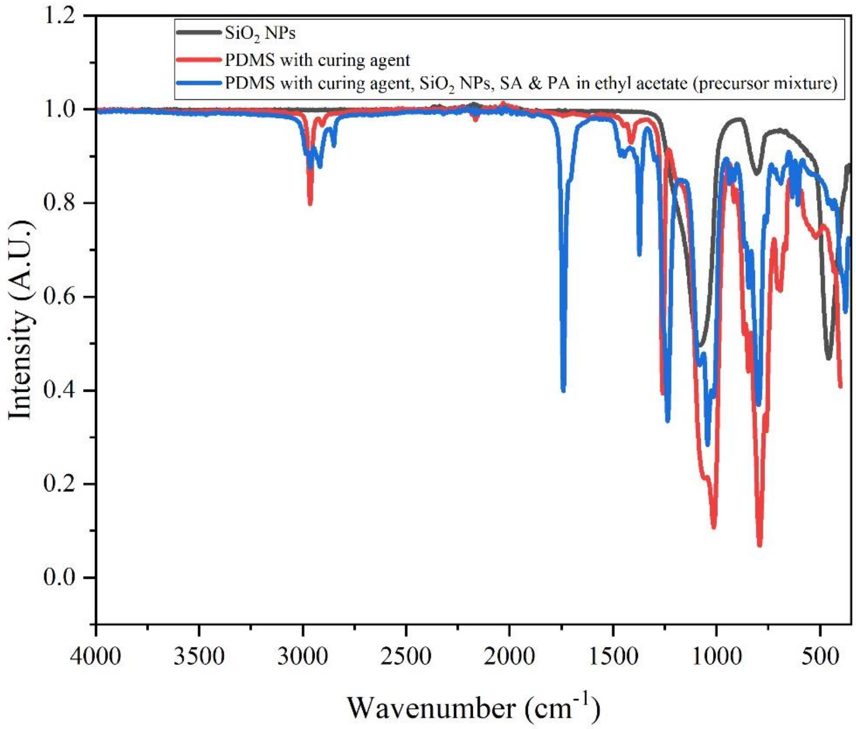

Figure S3. FT-IR spectrum of some of the starting materials and precursor mixture (in ethyl acetate), used to deposit film **PDMS/SiO_2/SA+PA.**

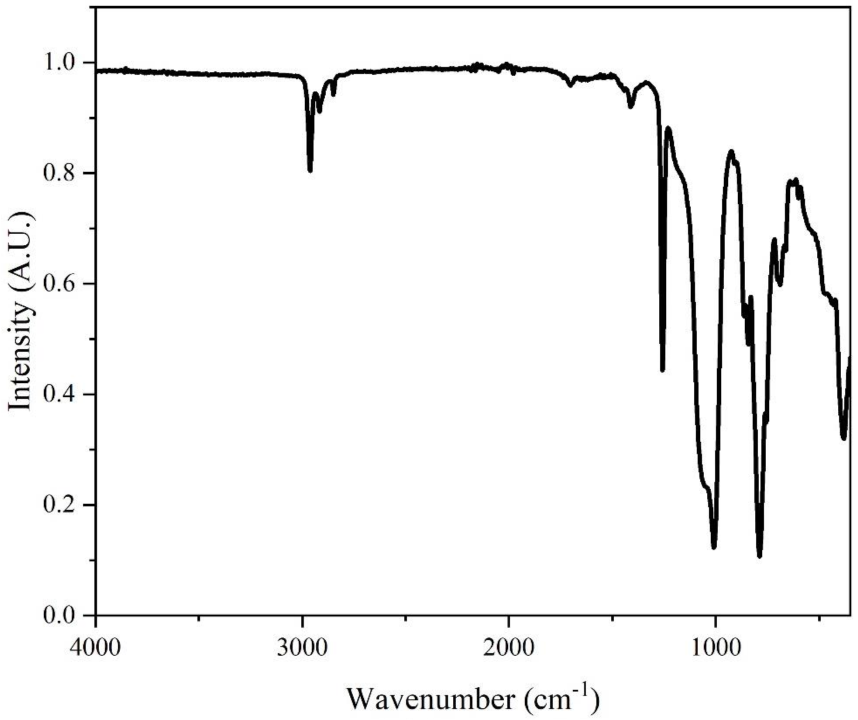

Figure S4. FT-IR spectrum of film **PDMS/SiO_2/FAS.**

(a) (b) (c)

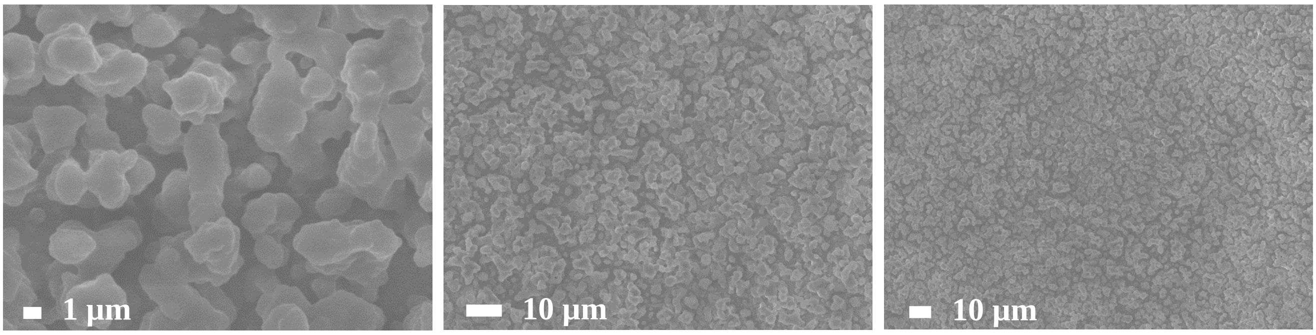

Figure S5. Images a – c are SEM images of **0.5PDMS/SiO_2/0.5FAS/40 (**SiO_2 NPs coated with 1H,1H,2H,2H-perfluorooctyltriethoxysilane (FAS C_8), deposited at 40 min).
